# An assessment of nicotine pharmacokinetics and subjective effects of the pulze heated tobacco system compared with cigarettes

**DOI:** 10.1038/s41598-023-36259-1

**Published:** 2023-06-03

**Authors:** Simon McDermott, Kerstin Reichmann, Elizabeth Mason, Ian M. Fearon, Grant O’Connell, Thomas Nahde

**Affiliations:** 1 ICON PLC, South County Business Park, Leopardstown, Dublin 18, Ireland; 2 Imperial Brands PLC, 121 Winterstoke Road, Bristol, BS3 2LL UK; 3 whatIF? Consulting Ltd, The Crispin, Burr Street, Harwell, OX11 0DT UK; 4 Reemtsma Cigarettenfabriken GmbH, Max-Born-Straße 4, 22761 Hamburg, Germany

**Keywords:** Social behaviour, Biomarkers, Medical research, Risk factors

## Abstract

Nicotine delivery and subjective effects are determinants of the ability of potentially less harmful tobacco products such as heated tobacco products (HTPs) to support adult smokers in switching away from cigarettes, and therefore to support tobacco harm reduction. This open-label, randomised, crossover, clinical study in 24 healthy adult smokers study assessed nicotine pharmacokinetics and subjective effects of the Pulze Heated Tobacco System (HTS; Pulze HTP device and three iD stick variants—Intense American Blend, Regular American Blend and Regular Menthol) compared with subjects’ usual brand cigarettes (UBC). C_max_ and AUC_t_ were highest for UBC and significantly lower for each Pulze HTS variant. C_max_ and AUC_t_ were significantly higher for Intense American Blend compared with Regular American Blend, while AUC_t_ was significantly higher for Intense American Blend compared with Regular Menthol. Median T_max_ was lowest (i.e., nicotine delivery was fastest) for subjects’ usual brand cigarettes and similar across the iD stick variants, although no between-product differences were statistically significant. All study products reduced urges to smoke; this effect was greatest for cigarettes although this was not statistically significant. Product evaluation scores for each Pulze HTS variant in the domains of ‘satisfaction’, ‘psychological reward’ and ‘relief’ were similar, and lower than those for UBC. These data demonstrate that the Pulze HTS effectively delivers nicotine and generates positive subjective effects, including satisfaction and reduced urge to smoke. This supports the conclusion that the Pulze HTS may be an acceptable alternative to cigarettes for adult smokers while having a lower abuse liability than cigarettes.

## Introduction

Cigarette smoking is a cause of serious diseases including lung cancer, heart disease, and emphysema, and is a leading cause of preventable deaths^1–4^. Globally, smoking is reported to be responsible for more than 7 million deaths per year^4^. In Europe, although smoking prevalence is declining smoking still accounts for up to 25% of all-cause mortality^5^ and leads to approximately 700,000 deaths each year^6^. While nicotine in cigarette smoke is not harmless, it is not the primary cause of the harmful effects of cigarette smoking^7^. Instead, smoking-related harms are caused by smokers inhaling chemical toxicants which are formed during the processes of tobacco combustion and pyrolysis^8^. Around 7000 individual chemicals have been identified in cigarette smoke^9^ and many of these are linked to cardiovascular disease, respiratory disease, lung cancer and reproductive/developmental toxicity^10^. Stopping smoking eliminates exposure to associated toxicants and conveys the greatest possible reduction in disease risk for smokers, and is therefore the best course of action smokers can take to improve their health^2^. However, while large proportions of smokers report intending to quit smoking only a small percentage successfully stop smoking each year^11–13^.

In 2001 the US Institute of Medicine issued the report ‘Clearing the Smoke’, in which it was proposed that ‘For many diseases attributable to tobacco use, reducing risk of disease by reducing exposure to tobacco toxicants is feasible’^14,15^. This laid the foundation for tobacco harm reduction (THR), which relies on the fundamental principle that both the individual- and population-level health impacts of cigarette smoking can be reduced by the development of, and smoker access to, novel nicotine and tobacco products which deliver nicotine but in the reduced presence, or absence, of the chemicals responsible for smoking-related disease. Particularly aimed at those adult smokers who are either uninterested or unwilling to stop smoking^16^, support for a toxicant reduction approach to THR is growing. Many public health bodies including the UK Office for Health Improvement and Disparities (formerly Public Health England)^17^, the UK Royal College of Physicians^16^, the Government of Canada^18^, and the New Zealand Ministry of Health^19^, now advocate for a toxicant reduction approach to THR.

Heated tobacco products (HTPs) in general electrically heat tobacco to temperatures significantly lower than those which cause pyrolysis and combustion in combustible cigarettes^20–23^. This electrical heating causes the formation of an inhalable aerosol which contains nicotine^24^. However, aerosol from HTPs contains significantly fewer and lower levels of harmful chemicals than those found in cigarette smoke^25–29^. A number of clinical studies examining toxicant exposure have demonstrated significantly reduced exposure to toxicants linked to cardiovascular disease, respiratory disease, and reproductive/developmental toxicity, in smokers who switch to using HTPs^30–38^. Furthermore, these reduced toxicant exposures in switching smokers are associated with favourable changes in biomarkers of potential harm including those indicative of risk of cardiovascular disease, respiratory disease, inflammation/oxidative stress, and lung cancer^31,33,39–42^. This provides evidence of the significant harm reduction potential of HTPs and of the role they can play in making a meaningful contribution to THR strategies. However, reduced toxicant exposure is only one attribute that can determine the harm reduction potential of HTPs. Importantly, if the harm reduction potential of a HTP is to be maximised, reduced exposure must be allied with HTP uptake by sufficient numbers of adult smokers who would otherwise continue to smoke cigarettes. It has been suggested for smoking alternatives, such as electronic nicotine delivery systems (ENDS) and HTPs, that nicotine delivery and subjective effects such as satisfaction, liking and reductions in withdrawal symptoms are important factors, alongside their ability to replicate some of the ritualistic/sensorial cues associated with cigarette smoking, in determining their ability to facilitate smokers’ switching away from smoking^43–48^. In support of the association between nicotine delivery, switching, and harm reduction, increasing the e-liquid nicotine concentration of an ENDS is associated both with increased nicotine delivery^44^ and higher rates of switching away from cigarette smoking^49^.

Reductions in toxicant exposure and ensuing changes in disease risk in smokers switching to using HTPs have been well described in the literature. However, despite the importance of nicotine delivery, along with positive subjective effects, in providing an acceptable alternative to cigarettes for adult smokers and therefore in supporting THR, much less is known about the delivery of nicotine from commercially available HTPs. The Pulze Heated Tobacco System (HTS)*,* which comprises the Pulze HTP device and iD sticks, is a novel tobacco product which electrically heats a consumable stick that contains reconstituted tobacco to a mean maximum internal temperature of 245 °C, substantially below the point of tobacco combustion. Due to the low operating temperature of the Pulze HTS device, emissions levels of a number of toxicants are significantly lower than those found in cigarette smoke^25^, although these findings are from an earlier, prototype version of the iD sticks. In this paper, findings are presented from an assessment of nicotine pharmacokinetics and subjective effects of three different variants (Intense American Blend, Regular American Blend and Regular Menthol) of iD sticks used with the Pulze HTS. These data are used to assess whether these attributes contribute to the likely acceptability of the Pulze HTS among adult smokers and therefore to establish the THR potential of the Pulze HTS.

## Results

### Subject demographics

Brief demographic details of the 24 subjects in the safety population are provided in Table 1, both for each randomisation sequence and overall. Fifty-eight percent of subjects were male, and all subjects were white and not of Hispanic/Latino origin. No major differences in demographics were seen between the randomisation sequence groups although the mean age and number of years smoking of the subjects in one of the product sequence groups was lower than in the other 3 groups (Table 1). Of the 24 subjects, 23 smoked non-menthol cigarettes as their usual brand of cigarette and a single subject smoked menthol cigarettes.

**Table 1:** Summary of subject demographics.

Trait	n	Randomised product sequence^a^				Overall
		ABCD	BDAC	CADB	DCBA	
		6	6	6	6	24
Sex	Female	2 (33%)	4 (67%)	2 (33%)	2 (33%)	10 (42%)
	Male	4 (67%)	2 (33%)	4 (67%)	4 (67%)	14 (58%)
Race	White	6 (100%)	6 (100%)	6 (100%)	6 (100%)	24 (100%)
Ethnicity	Not Hispanic or Latino	6 (100%)	6 (100%)	6 (100%)	6 (100%)	24 (100%)
Age (years)	Mean	40.7	31.8	49.2	42.2	41.0
	SD	12.75	8.11	7.14	12.50	11.59
	Minimum	24	25	40	21	21
	Median	37.5	29.5	51.0	44.0	39.0
	Maximum	61	45	56	54	61
Weight (kg)	Mean	70.38	68.20	71.67	70.15	70.10
	SD	8.765	12.529	5.814	10.344	9.113
	Minim	58.4	52.5	64.4	56.3	52.5
	Median	70.90	69.20	70.90	69.45	69.80
	Maximum	81.0	82.0	78.2	87.0	87.0
Height (cm)	Mean	173.0	164.5	171.2	167.3	169.0
	SD	8.29	8.09	4.79	6.12	7.33
	Minimum	161	154	167	162	154
	Median	175.0	162.5	169.5	165.5	167.5
	Maximum	183	176	178	179	183
BMI (kg/m^2^)	Mean	23.647	25.057	24.503	25.010	24.554
	SD	3.7250	3.0722	2.2506	2.9411	2.8958
	Minimum	18.87	19.76	20.33	20.19	18.87
	Median	22.945	25.980	24.835	25.640	24.835
	Maximum	29.39	27.93	26.67	27.59	29.39
Cigarettes smoked per day	Mean	20.7	19.0	22.3	22.3	21.1
	SD	4.08	5.48	5.16	5.16	4.87
	Minimum	19	14	19	19	14
	Median	19.0	19.0	19.0	19.0	19.0
	Maximum	29	29	29	29	29
Number of years smoking cigarettes	Mean	22.50	13.00	21.67	20.50	19.42
	SD	11.005	9.274	4.274	9.793	9.203
	Minimum	4.0	1.0	17.0	7.0	1.0
	Median	22.50	11.50	22.00	17.50	19.50
	Maximum	37.0	27.0	27.0	33.0	37.0

^a^Individual product codes were Pulze HTS used with (A) iD Intense American Blend sticks, (B) iD Regular American Blend sticks and (C) iD Regular Menthol sticks, and (D) subjects’ usual brand cigarettes.

### Nicotine pharmacokinetics

**Figure 1:**
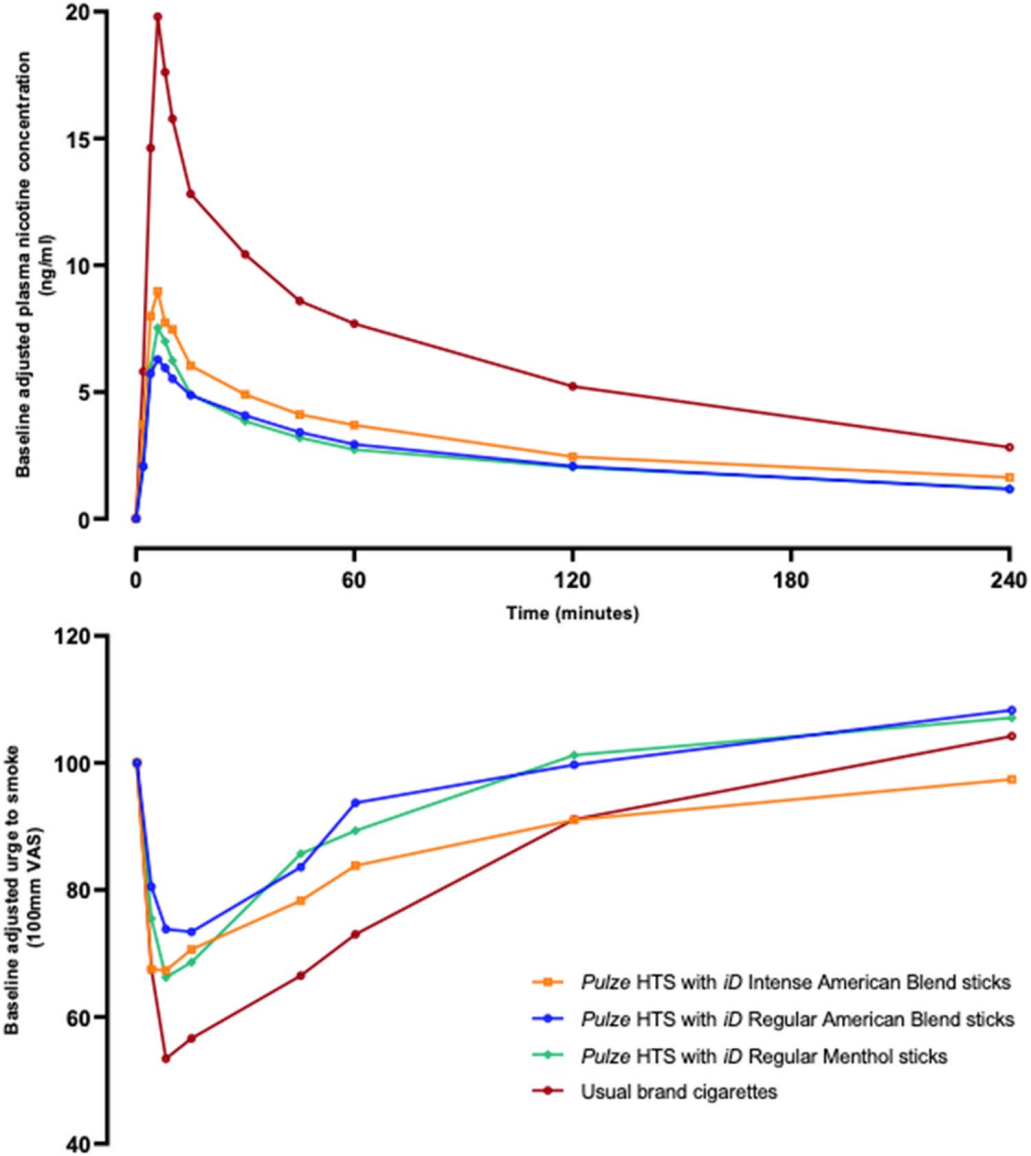
Baseline-adjusted plasma nicotine concentrations (upper) and urge to smoke (lower) over time following controlled product use in the outcomes population. N = 23–24 in each case. Data points are arithmetic means for each product at each timepoint. Error bars have been removed for clarity; for variability estimates refer to Tables 2 and 4. *VAS* visual analog scale, *HTS* heated tobacco system.

Prior to the start of the controlled puffing sessions and following 12 h of abstinence from the use of any tobacco- or nicotine-containing products, the mean uncorrected plasma nicotine concentration was 1.13 ng/ml (standard deviation [SD] 0.773 ng/ml, minimum 0.1 ng/ml, maximum 4.56 ng/ml, median 0.987 ng/ml). During use of any of the study products in the controlled use sessions (puffs taken at 30-s intervals with puffs 3 s in duration), plasma nicotine levels rose rapidly (Fig. 1). On average, during the controlled use session subjects took between 8.3 and 8.5 puffs on each of the iD stick variants and 9.8 puffs on their usual brand cigarettes (Supplementary Table 1).

The mean maximum plasma nicotine concentration (C_max_) and AUC values were highest for usual brand cigarettes and lower for each of the iD stick variants used with the Pulze HTS (Intense American Blend, Regular American Blend and Regular Menthol; Table 2); statistical analyses showed that C_max_ and AUC_t_ for each Pulze HTS variant were significantly lower than for subjects’ usual brand cigarettes (Table 3). Furthermore, C_max_ and AUC_t_ were significantly higher for Intense American Blend compared with Regular American Blend, while AUC_t_ (but not C_max_) was significantly higher for Intense American Blend compared with Regular Menthol. No significant differences were seen for either C_max_ or AUC_t_ between Regular American Blend and Regular Menthol (Table 3).

**Table 2:** Baseline-adjusted plasma nicotine pharmacokinetic parameters following controlled product use in the outcomes population.

Study product	Variable	C_max_ (ng/ml)	AUC_0–90_ (ng*min/ml)	AUC_0–240_ (ng*min/ml)	AUC_t_ (ng*min/ml)	T_max_ (mins)
Pulze HTS with iD intense American blend sticks	n	23	24	23	23	23
	Geometric mean	8.025	353.5	690.5	690.9	NC
	Geometric CV (%)	88.7	67.6	44.9	44.9	NC
	Mean	10.50	407.7	745.5	746.0	7.456
	SD	8.2842	188.97	271.18	271.24	3.3963
	Median	5.821	398.1	763.5	763.1	6.000
	Range	1.29, 36.1	70.2, 761	198, 1220	198, 1220	2.37, 15.08
Pulze HTS with iD regular American blend sticks	n	24	24	24	24	24
	Geometric mean	6.203	296.3	546.3	546.7	NC
	Geometric CV (%)	79.9	52.9	44.7	44.7	NC
	Mean	7.539	322.9	586.1	586.5	11.678
	SD	4.6531	110.19	191.85	192.03	11.1368
	Median	5.508	334.6	569.7	570.8	7.084
	Range	0.59, 18.1	44.9, 539	125, 944	125, 946	4.00, 44.88
Pulze HTS with iD regular menthol sticks	n	24	24	24	24	24
	Geometric mean	7.074	300.0	548.1	548.1	NC
	Geometric CV (%)	61.8	43.2	41.3	41.3	NC
	Mean	8.191	321.1	585.1	585.0	8.692
	SD	4.4465	104.21	191.32	191.16	5.6207
	Median	7.179	348.9	590.1	590.3	6.942
	Range	2.45, 18.0	(83.4, 513	179, 888	179, 888	4.00, 29.88
Usual brand cigarettes	n	23	23	23	23	23
	Geometric Mean	19.24	826.6	1466	1467	NC
	Geometric CV (%)	54.7	28.1	27.2	27.2	NC
	Mean	21.68	853.6	1513	1514	9.059
	SD	10.810	200.39	368.80	368.91	8.1994
	Median	20.07	912.9	1501	1501	6.250
	Range	6.50, 51.4	(343, 1260	(19, 2180	719, 2180	4.03, 44.90

*C*_*max*_ maximum plasma nicotine concentration, *AUC*_*0–90*_* and AUC*_*0–240*_ area under the plasma nicotine concentration–time curve from zero to 90 and 240 min, respectively, *AUC*_*t*_ area under the plasma nicotine concentration–time curve from zero to the time of the last measurable non-zero concentration, *T*_*max*_ time of the maximum plasma nicotine concentration, *NC* not calculated, *n* number of observations used in the analysis, *CV* coefficient of variation, *SD* standard deviation, *HTS* heated tobacco system.

**Table 3:** Summary of statistical comparisons of baseline-adjusted plasma nicotine pharmacokinetic parameters C_max_ and AUC_t_ in the outcomes population.

Product comparison	Parameter	Geometric LS means		% Geometric LS mean ratio (test/reference)	95% CI	p value
		Test (n)	Reference (n)			
iD intense American blend sticks versus usual brand cigarettes	C_max_	7.931 (23)	19.53 (23)	40.62	31.80–51.88	< .0001*
	AUC_t_	694.8 (23)	1475 (23)	47.11	40.27–55.11	< .0001*
iD regular American blend sticks versus usual brand cigarettes	C_max_	6.203 (24)	19.53 (23)	31.77	24.96–40.43	< .0001*
	AUC_t_	546.7 (24)	1475 (23)	37.07	31.76–43.26	< .0001*
iD regular menthol sticks versus usual brand cigarettes	C_max_	7.074 (24)	19.53 (23)	36.23	28.47–46.11	< .0001*
	AUC_t_	548.1 (24)	1475 (23)	37.17	31.85–43.38	< .0001*
iD intense American blend sticks versus iD regular American blend sticks	C_max_	7.931 (23)	6.203 (24)	127.85	100.46–162.71	0.0459*
	AUC_t_	694.8 (23)	546.7 (24)	127.08	108.89–148.32	0.0029*
iD intense American blend sticks versus iD regular menthol sticks	C_max_	7.931 (23)	7.074 (24)	112.11	88.09–142.68	0.3473
	AUC_t_	694.8 (23)	548.1 (24)	126.75	108.60–147.93	0.0032*
iD regular American blend sticks versus iD regular menthol sticks	C_max_	6.203 (24)	7.074 (24)	87.69	69.14–111.21	0.2735
	AUC_t_	546.7 (24)	548.1 (24)	99.74	85.65–116.15	0.9728

The mixed model included sequence, product, and study period as fixed effects and subject-nested-within-sequence as a random effect. Mixed model with a default (variance component) covariance structure was used. Parameters were ln-transformed prior to analysis. Geometric LS means were calculated by exponentiating the LS Means from the ANOVA. *C*_*max*_ maximum plasma nicotine concentration, *AUC*_*t*_ area under the plasma nicotine concentration–time curve from zero to the time of the last measurable non-zero concentration, *LS* least-squares, *CI* confidence interval, *n* number of observations.

Among the Pulze HTS iD stick variants, the median time taken to reach the maximum plasma nicotine concentration (T_max_; Table 2) was highest for Regular American Blend (7.084 min), slightly lower for Regular Menthol (6.942 min), and lowest for Intense American Blend (6.000 min). Median T_max_ for subjects’ usual brand cigarettes was 6.250 min (Table 2). Statistical analyses of differences between study products for T_max_ values are provided in Supplementary Table 2; differences between products were not statistically significant for any comparisons.

### Subjective effects

Urge to smoke a cigarette was assessed prior to study product use and at various timepoints after product use using a single-item questionnaire with responses from subjects to the question ‘How strong is your urge to smoke right now?’ provided on a 100 mm VAS ranging from ‘Not at all’ to ‘Extreme’. Mean baseline (prior to study product use) VAS scores ranged from 57.8 to 64.2 for all products, and descriptive data for mean VAS scores following product use are presented in Table 4. Use of all study products elicited robust reductions in urge to smoke (Fig. 1). The mean maximum change in urge to smoke (E_max_) was highest for subjects’ usual brand cigarettes (49.8 ± 30.10) and approximately 20–30% lower for each of the Pulze HTS variants (range 32.9 ± 27.13 to 40.3 ± 25.75). However, statistically significant differences in E_max_ values between products were only seen for the comparison of Regular American Blend and subjects’ usual brand cigarettes and not for any other comparisons (Table 5). The time of the maximum change in urge to smoke (TE_max_) was similar across all study products, while mean AUEC_0–240_ (area under the effect-time curve from zero to 240 min) was highest for subjects’ usual brand cigarettes, lower for Intense American Blend and lowest for Regular American Blend and Regular Menthol (Table 4). However, no statistically significant differences in AUEC_0–240_ were seen between the individual study products (Table 5).

**Table 4:** Summary of urge to smoke parameters following controlled product use in the outcomes population.

Parameter	Pulze HTS with iD intense American blend sticks	Pulze HTS with iD regular American blend sticks	Pulze HTS with iD regular menthol sticks	Usual brand cigarettes
E_max_				
n	23	23	24	22
Mean ± SD	40.3 ± 25.75	32.9 ± 27.13	39.7 ± 29.72	49.8 ± 30.10
Median	30.0	25.0	37.0	45.5
95% CI	29.1, 51.4	21.2, 44.6	27.2, 52.3	36.5, 63.2
TE_max_				
n	23	23	24	22
Mean ± SD	13.543 ± 16.6848	18.120 ± 26.4994	15.248 ± 18.9891	15.758 ± 16.2960
Median	7.017	7.083	7.042	7.250
95% CI	6.328, 20.758	6.661, 29.579	7.230, 23.267	8.532, 22.983
AUEC_0–240_				
n	23	23	24	22
Mean ± SD	2884 ± 5250.0	838.1 ± 5287.9	1048 ± 6427.9	3495 ± 5448.7
Median	2251	1089	1124	3298
95% CI	614.0, 5154	-1449, 3125	-1666, 3763	1079, 5911

*E*_*max*_ maximum value of the difference between pre- and post-use, *TEmax* time of the maximum difference between pre- and post-use, *AUEC*_*0–240*_ area under the effect-time curve from zero to 240 min, *n* number of observations, *CI* confidential interval, *SD* standard deviation, *HTS* heated tobacco system.

**Table 5:** Summary of statistical comparisons of urge to smoke parameters E_max_ and AUEC_0–240_ in the outcomes population.

Product comparison	Parameter	LS Means		LS means difference (test–reference)	95% CI	p value
		Test (n)	Reference (n)			
iD intense American blend sticks versus usual brand cigarettes	E_max_	39.70 (23)	50.71 (22)	− 11.00	− 23.14, 1.13	0.0747
	AUEC_0-240_	2917 (23)	3575 (22)	− 658.1	− 3649.36, 2333.10	0.6617
iD regular American blend sticks versus usual brand cigarettes	E_max_	33.63 (23)	50.71 (22)	− 17.07	− 29.20, − 4.94	0.0065
	AUEC_0-240_	859.9 (23)	3575 (22)	− 2715	− 5704.42, 274.90	0.0744
iD regular menthol sticks versus usual brand cigarettes	E_max_	39.71 (24)	50.71 (22)	− 11.00	− 22.95, 0.95	0.0707
	AUEC_0-240_	1048 (24)	3575 (22)	− 2526	− 5475.16, 422.75	0.0918
iD intense American blend sticks versus iD regular American blend sticks	E_max_	39.70 (23)	33.63 (23)	6.071	− 5.86, 18.00	0.3131
	AUEC_0-240_	2917 (23)	859.9 (23)	2057	− 888.02, 5001.27	0.1677
iD intense American blend sticks versus iD regular menthol sticks	E_max_	39.70 (23)	39.71 (24)	− 0.006731	− 11.78, 11.77	0.9991
	AUEC_0-240_	2917 (23)	1048 (24)	1868	− 1040.26, 4776.41	0.2040
iD regular American blend sticks versus iD regular menthol sticks	E_max_	33.63 (23)	39.71 (24)	− 6.077	− 17.85, 5.70	0.3063
	AUEC_0-240_	859.9 (23)	1048 (24)	− 188.6	− 3096.85, 2719.75	0.8973

The mixed model includes product sequence, period, and product as fixed effects and subject nested within product sequence as a random effect. Mixed model with a default (variance component) covariance structure was used. Least-squares means (LS Means) were calculated from the ANOVA. *E*_*max*_ maximum change in urge to smoke, *AUEC*_*0–240*_ area under the effect-time curve from zero to the 240 min, *LS* least-squares, *CI* confidence interval, *n* number of observations.

PES scores are presented in Table 6. On a scale of 1 to 7, with 1 being “not at all” and 7 being “extremely”, overall mean product evaluation scores for each of the Pulze HTS variants in the domain of ‘satisfaction’ ranged from 2.49 to 3.18, with a slightly higher score of 4.43 for subjects’ usual brand cigarettes. Similarly, in the domains of psychological reward, relief, ease of use, and comfort using in public, mean values were highest for subjects’ usual brand cigarettes and slightly lower, and comparable, for each of the Pulze HTS variants (Table 6). For aversion, mean values were highest for subjects’ usual brand cigarettes and Intense American Blend and lower for both Regular American Blend and Regular Menthol. For dependence concerns, the highest mean value was for usual brand cigarettes and approximately 50% lower, and comparable, for each of the Pulze HTS variants.

**Table 6:** Summary of product evaluation scale factor scores by study product in the outcomes population.

Subscale	Pulze HTS with iD intense American blend sticks	Pulze HTS with iD regular American blend sticks	Pulze HTS with iD regular menthol sticks	Usual brand cigarettes
Satisfaction				
n	24	24	24	23
Mean ± SD	2.49 ± 1.250	2.55 ± 1.023	3.18 ± 1.451	4.43 ± 1.431
Median	2.55	2.50	3.15	4.00
95% CI	1.96, 3.02	2.11, 2.98	2.57, 3.80	3.81, 5.04
Psychological reward				
n	24	24	24	23
Mean ± SD	2.50 ± 1.169	2.32 ± 1.016	2.63 ± 1.235	3.56 ± 1.462
Median	2.50	2.10	2.30	3.20
95% CI	2.01, 2.99	1.89, 2.75	2.10, 3.15	2.92, 4.19
Aversion				
n	24	24	24	23
Mean ± SD	2.18 ± 1.353	1.68 ± 0.858	1.52 ± 0.708	2.17 ± 1.254
Median	2.00	1.40	1.00	1.80
95% CI	1.61, 2.75	1.32, 2.04	1.22, 1.82	1.63, 2.72
Relief				
n	24	24	24	23
Mean ± SD	3.78 ± 1.358	3.55 ± 1.175	3.68 ± 1.150	4.57 ± 1.293
Median	3.80	3.70	3.80	4.40
95% CI	3.21, 4.36	3.05, 4.05	3.20, 4.17	4.01, 5.12
Was it easy to use?				
n	24	24	24	23
Mean ± SD	5.2 ± 1.67	5.3 ± 1.86	5.3 ± 1.54	5.9 ± 1.24
Median	5.0	6.0	5.0	7.0
95% CI	4.5, 5.9	4.5, 6.1	4.6, 5.9	5.4, 6.4
Comfortable using the product in public?				
n	24	24	24	23
Mean ± SD	4.7 ± 1.83	4.9 ± 1.96	5.4 ± 1.50	5.7 ± 1.56
Median	5.0	5.0	6.0	6.0
95% CI	3.9, 5.4	4.0, 5.7	4.7, 6.0	5.0, 6.3
Concerned you would become dependent?				
n	24	24	24	23
Mean ± SD	1.5 ± 1.02	1.6 ± 1.31	1.5 ± 0.78	3.3 ± 2.39
Median	1.0	1.0	1.0	2.0
95% CI	1.1, 2.0	1.1, 2.2	1.1, 1.8	2.3, 4.4

Subscale scores in the domains of satisfaction (items 1, 2, 3, and 12), psychological reward (items 4, 5, 6, 7, and 8), aversion (items 9, 10, 16, and 18), and relief (items 11, 13, 14, 15, and reversed for 20), were generated from the individual items as described previously^55^. *n* number of observations, *SD* standard deviation, *CI* confidence intervals, *HTS* heated tobacco system.

Regarding intent to use the product again, 13%, 17%, and 13% of subjects expressed positive likelihood (assessed as a rating on the VAS between the mid-point and ‘definitely would’) of using the Pulze HTS with the iD Intense American Blend stick, the iD Regular American Blend stick, and the iD Regular Menthol stick again, respectively, compared with 48% of subjects who expressed positive likelihood of using their usual brand cigarettes again. Overall, mean raw VAS scores for intent to use the product again ranged from 25.0 to 26.0 (0 = definitely would not and 100 = definitely would) for the Pulze HTS variants, with a higher score of 51.3 for subjects’ usual brand cigarettes.

### Safety

There were no SAEs reported in this study and no subjects were discontinued due to AEs. During the product trial period on Day −1, during which subjects were allowed to use a single iD of their choice with the Pulze HTS, one mild AE (dizziness) was reported by one (4%) subject. The Investigator considered this event unrelated to study product use. Overall, AEs were infrequently reported in this study, with six AEs reported by five (21%) subjects after study product randomisation. Catheter site pain was reported three times by three (13%) subjects, and the remaining AEs (constipation, dizziness, and neck pain) were reported by one (4%) subject each. The constipation and dizziness events were moderate in severity, and the catheter site pain and neck pain events were mild. The Investigator considered all AEs to be unlikely related or unrelated to study product.

Mean vital signs (heart rate and blood pressures) remained within normal limits at all study time points, with minimal change from baseline. There were no individual clinically significant vital sign findings in this study.

## Discussion

The cornerstone of THR is the principle that the harms associated with cigarette smoking, at both the individual and population levels, can be reduced by providing adult smokers with alternatives to cigarettes which deliver nicotine but with lower levels of the harmful chemicals found in cigarette smoke^14–16^. Acceptability of novel tobacco and nicotine products to adult smokers is an important driver of the likelihood that a given product will support smokers, who would otherwise continue to smoke, in transitioning away from cigarette smoking. Acceptability itself is driven by two main factors; the first factor is nicotine delivery, and it is generally considered that more cigarette-like nicotine delivery is important to uptake of novel tobacco products including ENDS and HTPs, their continued use, and prevention of relapse back to cigarette smoking^43–47^. Secondly, subjective effects contribute strongly to acceptability. Subjective effects may include positive effects such as satisfaction and liking^50,51^, as well as relief from negative effects such as urges to smoke/cigarette cravings^52^ and withdrawal symptoms^53^. Given the centrality therefore of nicotine delivery and subjective effects in determining the THR potential of non-combustible tobacco products including HTPs, this paper reports a clinical study designed to assess nicotine pharmacokinetics and various subjective effects measures in adult smokers when they used three different variants of iD tobacco sticks with the Pulze HTS. Regarding nicotine pharmacokinetics, use of all three variants of iD sticks used with the Pulze HTS under controlled conditions elicited delivery of nicotine, and there was no difference in nicotine delivery between the comparable menthol and non-menthol (tobacco) iD stick variants. As may be expected, there was evidence of a dose–response effect with greater levels of nicotine in the blood plasma (in terms of both C_max_ and AUC) seen when subjects used iD Intense American Blend sticks (which have a higher per-stick aerosol nicotine yield) compared with both iD Regular American Blend sticks and iD Regular Menthol sticks; such a dose-dependent effect has been reported previously for other HTPs^54^. Nicotine delivery however was statistically significantly different, again in terms of C_max_ and AUC, than nicotine delivery from subjects’ usual brand of cigarettes. Median T_max_ was lowest (i.e., nicotine delivery was fastest) for subjects’ usual brand cigarettes and similar across the iD stick variants, although no between-product differences were statistically significant. It is notable however that there was a high degree of variability in T_max_ across all study products, and also that the median T_max_ values were similar across all study products.

Regarding subjective effects, use of each of the iD sticks used with the Pulze HTS elicited reductions in the urge to smoke cigarettes, which although approximately 20–30% lower in magnitude than that seen when subjects smoked cigarette were only significantly different to the cigarette for the iD Regular American Blend stick variant. The mean time-course (i.e., onset) of urge reductions was also similar across the study products, and similar to the onset of nicotine delivery the iD Intense American Blend stick variant elicited the most rapid reduction in urge to smoke. The composite positive subjective effects of satisfaction, psychological reward and relief on the PES^55^ were similar across the iD stick variants and slightly lower than those for usual brand cigarettes. Subjects, on average, found the iD Regular American Blend and Regular Menthol stick variants to be less aversive than their usual brand cigarettes, while the iD Intense American Blend stick variant was rated as equally aversive. Importantly, subjects reported much lower concerns that they would become dependent on the iD sticks than on their usual brand cigarettes.

There are few subjective effects assessments of HTPs reported in the literature with which to assess our findings against. However, previous studies^46,54,56^ have reported, similar to our study, robust reductions in urge to smoke in clinical study subjects using various different types of HTPs. In contrast to our findings though, bearing in mind that direct comparisons are challenging since different questionnaire approaches were used, the difference between product liking scores between cigarettes and HTPs in one of the prior studies^54^ were far greater than the differences in PES composite satisfaction scores between cigarettes and the Pulze HTS used in this study. This suggests that different HTPs with different heating characteristics and/or different tobacco stick compositions may elicit dissimilar subjective effects and therefore differentially provide support to different smokers switching away from cigarette smoking. Overall though, our findings and those of others in the published literature demonstrate that HTPs in general provide relief from urges to smoke and other withdrawal effects and also generate positive subjective effects, both of which may contribute to their ability to help smokers transition away from cigarette smoking and support THR.

The findings from this study can also be utilised to make an assessment of the relative abuse liability of the Pulze HTS. Abuse liability is a composite measure that can be determined from nicotine pharmacokinetic and subjective effects assessments, which together constitute an assessment of the abuse liability (dependence potential) of a tobacco or nicotine-containing product^43,57^. It has been proposed that the THR potential, when considering the ability of novel nicotine and tobacco products to reduce harms among adult current smokers, is optimal when appeal and dependence potential (abuse liability) are high and when and toxicity/harmfulness are low^7^. In this regard, possessing at least some degree of dependence potential is beneficial since it allows the novel product to compete with cigarettes and support switching away from cigarette smoking^7,43,58,59^. Conversely, too high an abuse liability or dependence potential may lead to the novel product posing an initiation or addiction risk among non-users of nicotine products, particularly among susceptible populations such as youth and young adults^58,59^. When considering the data presented in this paper, subjects usual brand cigarettes have a high abuse liability since cigarette smoking elicited high blood nicotine levels over a short period of time, as well as inducing strong subjective effects such as satisfaction and psychological reward. This is consistent with the literature which suggests that cigarettes have the highest abuse liability of any tobacco/nicotine product^7,60^. Each of the iD stick variants also delivered nicotine effectively, and with a speed of onset of nicotine delivery (another determinant of abuse liability)^61^ comparable to cigarettes, but the degree of delivery was significantly lower than that from cigarettes. Similarly, although the Pulze HTS variants reduced urges to smoke comparably to subjects’ usual brand cigarettes, positive subjective effects were lower in magnitude than those caused by cigarette smoking. Whilst this should not be interpreted that the Pulze HTS is not addictive, taken together, these data suggest that each variant of the Pulze HTS possesses a degree of abuse liability but that this abuse liability is lower than that of cigarettes. Furthermore, since the C_max_ was greater and T_max_ was shorter for the Pulze *HTS* compared with those pharmacokinetic parameters reported from studies on nicotine replacement therapy (NRT) gum^62–65^ and the Nicorette Inhalator^54^, likely the abuse liability of the Pulze HTS falls somewhere between cigarettes and NRT. This proposal is in line with the relative risk scale for nicotine-containing products^7^. In the context of THR, this finding supports that while the Pulze HTS may be an acceptable alternative to cigarette smoking that is satisfying to adult smokers and therefore may support switching, the lower abuse liability compared with cigarettes suggests that the Pulze HTS likely presents less of an initiation/addiction risk among non-users of nicotine than cigarettes. Such a conclusion is supported by survey data which demonstrate that HTP use among never smokers is very uncommon in countries where they are currently marketed^66^. In summary, the potential generation by the Pulze HTS of an ‘off-ramp’ away from cigarette smoking, while minimising ‘on-ramp’ to initiating tobacco/nicotine use, may serve to enhance the contribution the Pulze HTS can make to THR strategies.

The results presented in this paper should be considered within the context of a number of limitations. Firstly, this study assessed nicotine pharmacokinetics in smokers who used different variants of the Pulze HTS, and the findings were used to make assumptions regarding the potential ability of the Pulze HTS to act as a satisfactory alternative to cigarettes and therefore play a role in supporting THR. While we conclude that the pharmacokinetic and subjective effects profiles of the Pulze HTS will support its use as an acceptable alternative to smoking cigarettes, no data are presented concerning its actual ability to help smokers switch. However, data on other HTPs support our conclusions. For example, a recent study found that in adult smokers asked to switch to using the IQOS HTP, smokers initially substituted IQOS for 59% of their average daily cigarette consumption, and this increased to 87% by at the end of the 3-week study^67^. Furthermore, in a longer-term study with the IQOS HTP, half of study participants who were asked to switch from cigarette smoking to using IQOS were predominantly using IQOS as their tobacco product at 6 months^40^. For the glo HTP, biochemically-determined compliance with switching from smoking cigarettes to using glo was seen in 65% of study participants at the end of a 12-month study^33^. Notably, the nicotine pharmacokinetic parameter C_max_, which is influential in an assessment of abuse liability and of a novel tobacco product’s potential to displace cigarette smoking^43–47,61^, is similar between the Pulze HTS and other types of HTPs^54,68^. Taken together with these published data, our findings support the conclusion that the Pulze HTS will likely have a positive impact on THR by supporting either cigarette displacement, resulting in smoking reduction, or complete switching away from cigarette smoking. Studies are underway to ascertain the extent to which the Pulze HTS reduces cigarette consumption in smokers who adopt its use and will be reported in a future manuscript.

A further limitation is that this study assessed use of the Pulze HTS in a clinical environment using a controlled puffing regimen, at a single point in time, and with only a very brief familiarisation period. Although it has not been reported in the literature for HTPs, for other inhaled nicotine products such as ENDS, increases in C_max_ have been reported over time as users transition from initial use and become experienced users^69,70^. This suggests, at least for ENDS, that some degree of familiarisation or acclimatisation occurs subsequent to first use, and this may be secondary to users changing their puffing topography over time^70,71^. It is possible that acclimatisation similarly occurs for HTPs, and therefore that the nicotine pharmacokinetic parameters reported in this paper do not necessarily reflect those that would be seen in an accustomed Pulze HTS user. However, increases in C_max_ values over time for ENDS use, when assessed using similar methods as those used in this Pulze HTS study, have been reported as being less than 25%^69^. Therefore, our assessment of the abuse liability of the Pulze HTS relative to cigarettes would still remain valid even if similar levels of acclimatisation occurred for this product. Additionally, we have used data from a confined clinical study to make an assessment of the abuse liability of the Pulze HTS in users with limited familiarity of using the product and following the prescribed use of a single Pulze iD stick. While this experimental approach allows us to estimate the relative abuse liability of the different tobacco products assessed in the study, absolute abuse liability in the real world may be different than that reported from this clinical study.

In conclusion, our pharmacokinetic and subjective effects assessments of three iD stick variants when used with the Pulze HTS demonstrates effective nicotine delivery, generates positive subjective effects including satisfaction, psychological reward, and relief, and reduces urge to smoke. These findings support the conclusion that the Pulze HTS is likely to be an acceptable and satisfying alternative to cigarettes for adult smokers who would otherwise continue to smoke and support transitioning away from cigarettes, and therefore that the Pulze HTS has the potential to make a meaningful contribution to THR. Furthermore, when our findings are used to generate an abuse liability assessment, we conclude that the Pulze HTS has a lower abuse liability than cigarettes. The potential generation by the Pulze HTS of an ‘off-ramp’ away from cigarette smoking, while minimising an ‘on-ramp’ to initiating tobacco/nicotine use, likely serves to enhance its THR potential.

### Methods

## Overall study design

This open-label, randomised, crossover, clinical study received favourable opinion (equivalent to ethics approval) from the Office for Research Ethics Committees Northern Ireland (ORECNI) Health and Social Care Research Ethics Committee A (reference number 21/NI/0144) prior to study commencement. The study was performed in accordance with ethical principles set forth in the Declaration of Helsinki and was compliant with the principles and requirements of International Council for Harmonisation Good Clinical Practice (ICH-GCP), the European Union Clinical Trials Directive, and applicable local regulatory requirements. The study was performed at a single clinical site in Belfast, Northern Ireland (UK) and was registered in the ClinicalTrials.gov repository (NCT05459857). Twenty-four (24) male and female healthy adult smokers participated in this study. Subjects attended the clinic site on two separate occasions: a screening visit and a 5-day confinement period. After completing the study, a follow-up telephone call was conducted for all subjects no longer than 1 week after the end of their confinement period. All subjects provided written informed consent prior to the commencement of any study procedures, including screening assessments.

### Study subjects

In total, 51 subjects were screened for participation in this study; 27 subjects either failed screening or were not needed for study participation (i.e., they were study reserves but were not required to take part in the study). Of the 24 enrolled subjects, 23 completed the study; one subject withdrew from the study for personal reasons.

Inclusion criteria were that subjects were healthy adults aged 21–65 years inclusive at screening; self-reported smoking at least 10 manufactured combustible (menthol or non-menthol) cigarettes per day (CPD) for at least 12 months prior to screening; had a urine cotinine ≥ 500 ng/mL at screening; had an exhaled carbon monoxide (eCO) level > 10 parts per million (ppm) at screening; if female and of childbearing potential were using at least one approved form of contraception; if female and of non-childbearing potential had undergone a sterilisation procedure at least 6 months prior to check-in or was postmenopausal with amenorrhea (verified by measuring follicle-stimulating hormone (FSH) levels) for at least 1 year prior to check-in; if a non-vasectomised male agreed to use a condom with spermicide or abstain from intercourse for the duration of the study and extending up to 90 days post-study; if male agreed not to donate sperm for duration of the study and extending up to 90 days post-study; was willing comply with the requirements of the study, including a willingness to use the study HTPs; and provided voluntary consent to participate in this study, which was documented by signing of the signed informed consent form.

Subjects were not allowed to enter the study if any exclusion criteria were met. The main exclusion criteria were a history or presence of clinically significant disease or disorder that, in the opinion of the Investigator, would have jeopardised the safety of the subject or impacted the validity of the study results; had a clinically significant abnormal finding on the physical examination, medical history, vital signs, electrocardiogram, or clinical laboratory results; had an acute illness (e.g., upper respiratory tract infection, viral infection) requiring treatment within 14 days prior to Check-in; systolic blood pressure (BP) < 90 mmHg or > 150 mmHg, diastolic BP < 40 mmHg or > 95 mmHg, or heart rate (HR) < 40 bpm or > 99 bpm at screening; estimated creatinine clearance (using the Cockcroft Gault equation) < 70 ml/min at screening; used medications known to interact with cytochrome P450 2A6 within 3 months prior to check in and throughout the study; used inhalers to treat any medical condition within 3 months prior to check in and throughout the study; used prescription or over-the-counter bronchodilator medication (e.g., inhaled or oral β-agonists) for treatment of any illness within 12 months prior to check in and throughout the study; was allergic to or could not tolerate menthol flavouring agents; had used any prescription smoking cessation treatments, including, but not limited to, varenicline (Chantix®) or bupropion (Zyban®) within 3 months prior to check in; was planning to quit smoking during the study or within the next 3 months or was postponing a quit attempt in order to participate in the study; or had donated blood or blood products (including plasma), had significant blood loss, or received whole blood or a blood product transfusion within 90 days prior to check in.

### Study products

The Pulze HTS, which comprises the Pulze HTP device and iD sticks, generates a nicotine-containing aerosol by heating consumable sticks containing reconstituted tobacco using an electrically powered heating device as its heating source. This heating creates an inhalable aerosol to deliver nicotine via the lungs. The device can be operated in two different user-selected modes (‘standard’ and ‘eco’ modes) in which the device heats to temperatures of 345 °C and 315 °C, respectively. In this study, subjects only used the Pulze HTP in ‘standard’ mode. Once switched on the Pulze HTS device is operational for 4 min, which allows users to take approximately 8–9 puffs from a single iD stick.

The tobacco sticks contain a portion of reconstituted tobacco and other non-tobacco components. Three test iD consumables (reconstituted tobacco sticks used with the Pulze HTS device) were assessed in this study; Intense American Blend, Regular American Blend, and Regular Menthol. The aerosol from the Intense and Regular American Blend sticks has a tobacco aroma, while the aerosol from the Regular Menthol sticks has a menthol aroma due to the inclusion of a menthol-flavoured monoacetate filter at the mouth-end of the stick. The reconstituted tobacco in each of the Pulze iD consumables used in this study has a target specification of 4.6 mg of nicotine per stick, and different aerosol yields of nicotine are achieved by the use of different filters in each of the different iD consumables. When used under International Organization for Standardization (ISO) intense machine puffing conditions (55 ml bell-shaped puff over 2 s with a 30-s interpuff interval and with filter vents blocked)^72^, the Intense American Blend variant produces a higher yield of nicotine (mean yield 1.09 mg per stick) than either the Regular American Blend or Regular Menthol variants (mean yields both 0.64 mg per stick).

For use as a comparator product, all subjects provided their usual brand of cigarette.

### Randomisation

Subjects who completed the study screening assessments were assigned a unique randomisation identification number. Subsequently, each subject, based on the identification number, were assigned to use the study products according to one of four product sequences, which were prepared by Celerion, Inc. These sequences were ABCD, BDAC, CADB and DCBA where A is the Pulze HTS with iD Intense American Blend sticks, B is the Pulze HTS with iD Regular American Blend sticks, C is the Pulze HTS with iD Regular Menthol sticks, and D is subjects’ usual brand cigarettes.

### Study procedures

**Figure 2:**
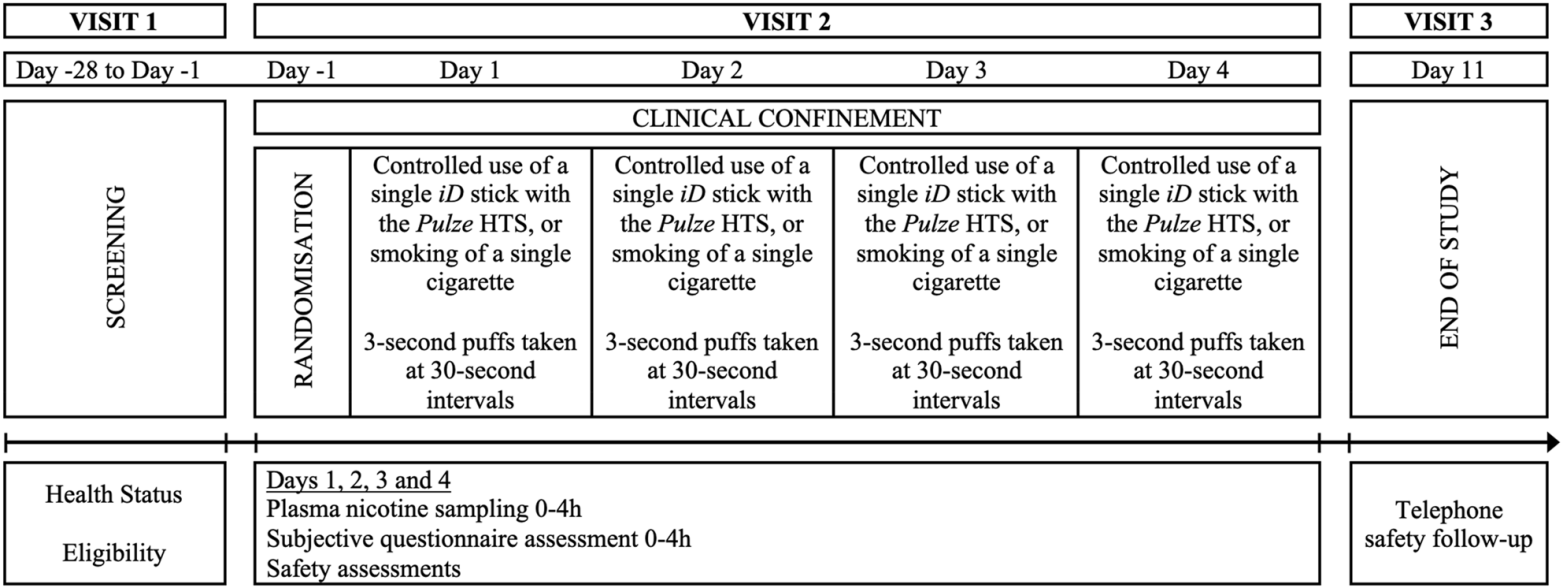
Study design overview.

This study was a randomised, open label, crossover, confinement study in 24 male and female cigarette smokers. The study assessed 3 test products and a cigarette comparator and evaluated nicotine pharmacokinetics, subjective effects, puff topography and product safety. An overview of the study design is presented in Fig. 2.

At Visit 1 (screening), which took place within 27 days prior to study procedures on Day −1, subjects underwent numerous assessments to check their eligibility to participate in the study, to review their health status, and to assess their nicotine consumption habits. Screening procedures included a physical examination (including oral cavity and oropharynx), vital signs, electrocardiogram, body mass index (BMI), clinical laboratory tests (hematology, serum chemistry, and urinalysis), serology, urine/saliva drug, urine/breath alcohol, cotinine screen, eCO, and pregnancy and FSH tests (for females as appropriate). If requested, subjects were offered smoking cessation advice and contact information for a smoking cessation support service.

Visit 2 was a 5-day confinement period; subjects who successfully completed the screening procedures and met all the inclusion criteria and none of the exclusion criteria were eligible to check in to the clinical site for the confinement period. Subjects checked into the clinic on Day −1 and remained at the clinic until Day 4 for daily study product use, nicotine pharmacokinetics sampling, subjective questionnaire assessments, puff topography evaluation, and safety assessments. On Day −1, following eligibility confirmation, subjects undertook a familiarisation session with the study HTPs and the questionnaires. The site’s clinical team explained how the Pulze HTS was to be used. Subjects had the opportunity to see the products/devices and packaging and participated in a product trial in which they consumed one iD stick*,* of a flavour chosen by each subject, using the Pulze HTS. An explanation of how the questionnaires were to be administered to the subjects was given. After the familiarisation session and completion of check-in procedures, subjects were allowed to smoke their own cigarettes ad libitum but abstained from the use of any tobacco- or nicotine-containing products for at least 12 h prior to the start of the controlled product use session on the morning of Day 1. In the morning of Day 1, after pre-use assessments and confirmation of eligibility, the subjects were randomised to 1 of 4 product sequences and then provided a single product of the study product in the sequence to which they had been randomised. On Days 1 through 4, subjects used the assigned study product under controlled conditions (i.e., completely used a single iD tobacco consumable HTP stick or smoked a single cigarette), with puffs taken at 30-s intervals and puffs 3 s in duration. Blood samples for nicotine assessment were collected 5 min prior to initiating product use and at 2, 4, 6, 8, 10, 15, 30, 45, 60, 120, and 240 min following the start of study product use. Subjective effects questionnaires were administered to the subjects at defined intervals throughout the day, and safety was also monitored throughout the day. For all subjects, meals and snacks were provided at the appropriate times during confinement at the clinic site. Each meal and/or snack served at the site was standardised, was similar in caloric content and composition, and was taken at approximately the same time on each day. When confined at the clinic site, subjects were required to fast from all food and drink except water between meals and snacks.

On Days 1 through 4, following the 4-h pharmacokinetic blood collection, subjects started a 4-h ad libitum product use session (no limits on cigarette or HTP consumption) with the same study product as that used during the morning controlled use session. Puff topography assessments were also made in the study but are not reported in this manuscript, and no blood samples were taken in this period for nicotine pharmacokinetic analyses. After completion of the ad libitum use session, subjects were allowed to smoke their own cigarettes ad libitum until at least 12 h prior to the start of the morning controlled product use session scheduled on the following day. On Day 4, following completion of study assessments, subjects were allowed to smoke their own cigarettes and left the clinic after completing all final check-out requirements.

A follow-up telephone call (Visit 3) was made by the clinic in an attempt to contact all subjects who used at least one study product (including subjects who terminated the study early) using their standard procedures approximately 7 days after the final product use to determine if any adverse events (AEs) had occurred since the last study visit.

### Nicotine pharmacokinetics

To determine blood plasma nicotine concentrations during and after use of the study products, blood samples (approximately 4 ml) were collected through an indwelling venous catheter at the time-points described above. Blood samples were drawn into dipotassium ethylenediaminetetraacetic acid (K_2_EDTA) vacutainer tubes via an intravenous catheter port and the plasma fraction separated off by centrifugation and pipetting. Plasma nicotine was analysed by liquid chromatography-tandem mass spectrometry (LC–MS/MS) at Celerion Bioanalytical Services (Lincoln, Nebraska, USA) using a validated analytical method with appropriate quality controls according to the Food and Drug Administration (FDA) Guidance for Industry (Title 21 Code of Federal Regulations Part 58). Processing of samples was completed by a non-tobacco user. The lower limit of quantification of plasma nicotine using the analytical method was 0.2 ng/ml.

### Subjective effects assessments

The Intent to Use [100 mm visual analog scale (VAS)], Urge to Smoke (VAS), and Product Evaluation Scale (7-point scale^55^) questionnaires were completed using a computerised tablet device. All relevant software specific to the electronic questionnaires were provided by IVR Clinical Concepts (IVRCC; Saratoga Springs, New York, USA). The Urge to Smoke questionnaire was completed at Time 0 (pre-product use) and at 4, 8, 15, 45, 60, 120, and 240 min relative to the start of product use on Days 1, 2, 3, and 4. The Intent to Use questionnaire was completed at 240 min following the start of study product use on Days 1, 2, 3, and 4. The 21-item Production Evaluation Scale (PES) questionnaire was completed at 240 min following the start of study product use on each of Days 1, 2, 3, and 4. PES subscale scores in the domains of satisfaction (items 1, 2, 3, and 12), psychological reward (items 4, 5, 6, 7, and 8), aversion (items 9, 10, 16, and 18), and relief (items 11, 13, 14, 15, and reversed for 20), were generated from the individual items as described previously^55^.

### Statistical analyses

The safety population comprised all subjects who had successfully completed eligibility requirements after checking in to the clinic site and used at least one study product. The outcomes population was a subset of the safety population and consisted of subjects who used a study product and had evaluable nicotine pharmacokinetics or subjective effects data. This population was used in the summary and analysis of all data presented in this paper.

Due to this being the first study assessing nicotine pharmacokinetics and subjective effects of the Pulze HTS, no sample size calculations could be performed. However, a sample size of 24 subjects was deemed adequate to meet the study objectives and this is in line with similar study designs in the literature^46,56,68^.

### Demographics

Descriptive statistics are reported for continuous variables (age, weight, height, and BMI) and frequency counts were tabulated for categorical demographics variables (sex, ethnicity, and race). Descriptive statistics are also provided for smoking history variables (cigarettes smoked per day and number of years smoking).

### Nicotine pharmacokinetics

Unadjusted plasma nicotine concentrations that were below the limit of quantitation (BLQ) were set to one-half of the lower limit of quantitation (LLOQ) for the calculation of descriptive statistics. Individual plasma nicotine concentrations were adjusted for baseline nicotine levels (“baseline-adjusted”) and all pharmacokinetic parameters were calculated based on the adjusted concentrations. Baseline adjustment was performed by subtraction of the pre-existing nicotine concentration from each nicotine concentration obtained after test product administration in that period/day for each subject using the following equation:

$${\text{C}}_{{\text{t}}} \, = {\text{ C}}_{{{\text{t}}\;{\text{unadjusted}}}} {-} \, \left[ {{\text{C}}_{0} \cdot{\text{ e}}^{{ - {\text{Kel}}\cdot{\text{t1}}}} } \right]$$

where C_t_ is the adjusted concentration at time t, C_t unadjusted_ is the observed concentration at time t, C_0_ is the pre-product use concentration (−5 min), Kel = ln(2)/t½, t½ is 2 h (approximate nicotine half-life), t is the actual sampling time since product administration, and t1 is the actual sampling time since the time of the pre-product use sample. After correction for pre-product use values, negative values were assigned a value of zero and all other values obtained were reported as is even if these values were BLQ.

SAS® software (Version 9.4) was used for data presentation and summarisation including descriptive statistics, statistical analyses, summary tables, graphs, and data listings. Descriptive statistics were generated for plasma nicotine concentrations and nicotine pharmacokinetic parameters by study product for all subjects, including sample size (n), arithmetic mean (mean), SD, coefficient of variation (CV%), standard error of the mean (SEM), minimum, median, and maximum at each nominal time-point. In addition, geometric mean, and geometric CV%, are provided for the C_max_ (maximum plasma nicotine concentration) and AUC (area under the plasma nicotine concentration–time curve) parameters. Mean concentration–time profiles are presented on linear scales. Missing data were treated as missing, and no imputation was conducted.

A linear mixed-effects model for analysis of variance (ANOVA) was performed on the natural log-transformed pharmacokinetic parameters C_max_ and AUC_t_ following the morning product use session on each of Days 1, 2, 3, and 4. The model included sequence, product, and study period as fixed effects and subject-nested-within-sequence as a random effect. Geometric least-squares means (LSM), and 95% confidence intervals (CIs), are provided for the pharmacokinetic parameters C_max_ and AUC_t_ by study product. Geometric LSM ratios, 95% CIs of the geometric LSM ratios, and p-values are provided for the product comparisons of C_max_ and AUC_t_. The comparisons of interest included each of the products compared to each other. The above statistical analyses were performed using SAS® PROC MIXED.

A non-parametric analysis (Wilcoxon Signed Rank test) was performed for the comparisons of T_max_ (time to maximum plasma nicotine concentration) between each of the study products. The median difference and 95% CI of the difference are presented for each comparison. The CIs were constructed using Walsh Averages and the appropriate quantile of the Wilcoxon Signed Rank Test statistic.

### Subjective effects

#### Urge to smoke

The derived parameters E_max_ (maximum reduction in urge to smoke), TE_max_ (time of the maximum reduction in urge to smoke), and AUEC_0–240_ (area under the time-effect curve between time zero and 240 min), are listed by subject and summarised by product using descriptive statistics, including n, mean, SD, CV%, SEM, minimum, Q1, median, Q3, maximum, and 95% CI. A linear mixed-effects model for ANOVA was used to compare urge to smoke parameters without data transformation; the mixed model includes product sequence, period, and product as fixed effects and subject nested within product sequence as a random effect. LSM and 95% CIs are provided for the E_max_ and AUEC_0–240_ by product. LSM difference, 95% CIs of the LSM difference, and p-values are provided for the product comparisons for E_max_ and AUEC_0–240_. The comparisons of interest included each of the products compared to each other.

#### Product evaluation scale

Descriptive statistics for the composite (satisfaction, psychological reward, aversion and relief) and individual (ease of use, comfort using in public and dependence concerns) PES factor scores^55^ are provided by product.

#### Intent to use

Descriptive statistics for the VAS raw score and bipolar score are summarised.

#### Safety assessments

Safety was monitored through physical examination (symptom-driven), vital signs measurements, electrocardiograms, and clinical laboratory tests (serum chemistry, hematology, and urinalysis). Adverse event (AE) information was also collected throughout the study. AEs [including serious AEs (SAEs)] were recorded from the start of the first product used until the end-of-study telephone call. Severity/intensity were graded as mild, moderate, or severe, and AEs were also assessed as unlikely, possibly, or probably related to the study product by the investigator.

## Supplementary Information

Supplementary Tables.

## Data Availability

The datasets generated during and/or analysed during the current study are available from the corresponding author on reasonable request.
