# Supplementary material for: An assessment of nicotine pharmacokinetics and subjective effects of the pulze heated tobacco system compared with cigarettes
Source: Sci Rep. 2023 Jun 3;13:9037. doi: 10.1038/s41598-023-36259-1 (PMC10239516; doi:10.1038/s41598-023-36259-1)
Supplement: Supplementary file 1 — Supplementary Tables. [file 41598_2023_36259_MOESM1_ESM.docx]

**Supplementary Table 1. Summary of Product Use in the Controlled Puffing Sessions.**

| **Variable** | **Pulze HTS with iD Intense American Blend sticks** | **Pulze HTS with iD Regular American Blend sticks** | **Pulze HTS with iD Regular Menthol sticks** | **Usual brand cigarettes** |
| --- | --- | --- | --- | --- |
| n | 24 | 24 | 24 | 23 |
| Mean | 8.3 | 8.5 | 8.5 | 9.8 |
| SD | 1.08 | 1.02 | 0.93 | 1.83 |
| CV(%) | 13.1 | 12.1 | 11.0 | 18.6 |
| SEM | 0.22 | 0.21 | 0.19 | 0.38 |
| Minimum | 7 | 6 | 6 | 7 |
| Median | 8.0 | 8.5 | 8.0 | 10.0 |
| Maximum | 12 | 11 | 10 | 14 |
| 95% CI | 7.8 - 8.7 | 8. 0 - 8.9 | 8.1 - 8.9 | 9.0 - 10.6 |

Abbreviations: n, number of observations; SD, standard deviation; CV(%), coefficient of variation; CI, confidence intervals; HTS, Heated Tobacco System.

**Supplementary Table 2. Summary of Statistical Comparisons of Baseline-Adjusted Plasma Nicotine Pharmacokinetic T_max_ in the Outcomes Population.**

|  | **Median** | | **Difference (Test – Reference)** | | |
| --- | --- | --- | --- | --- | --- |
| **Product Comparison** | **Test** | **Reference** | **Median** | **95% CI** | **p value** |
| iD Intense American Blend sticks versus usual brand cigarettes | 6.00 | 6.17 | -0.18 | (-1.7085, 0.8750) | 0.3832 |
| iD Regular American Blend sticks versus usual brand cigarettes | 8.02 | 6.25 | 0.00 | (-0.8495, 3.8585) | 0.2128 |
| iD Regular Menthol sticks versus usual brand cigarettes | 7.77 | 6.25 | -0.10 | (-1.0665, 1.3085) | 0.7572 |
| iD Intense American Blend sticks versus iD Regular American Blend sticks | 6.00 | 6.15 | -0.10 | (-4.3250, 0.9500) | 0.2028 |
| iD Intense American Blend sticks versus iD Regular Menthol sticks | 6.00 | 6.12 | -0.80 | (-1.8500, 1.0000) | 0.3264 |
| iD Regular American Blend sticks versus iD Regular Menthol sticks | 7.08 | 6.94 | 0.08 | (-0.7665, 3.9335) | 0.3339 |

Non-parametric analysis (Wilcoxon Signed Rank test) was performed for the comparisons of T_max_ between each of the study products. The median difference and 95% CI of the difference are presented for each comparison. The CIs were constructed using Walsh Averages and the appropriate quantile of the Wilcoxon Signed Rank Test statistic.
